# Nlz1/Znf703 acts as a repressor of transcription

**DOI:** 10.1186/1471-213X-8-108

**Published:** 2008-11-12

**Authors:** Mako Nakamura, Seong-Kyu Choe, Alexander P Runko, Paul D Gardner, Charles G Sagerström

**Affiliations:** 1 Department of Biochemistry and Molecular Pharmacology, University of Massachusetts Medical School, Worcester, USA; 2 Department of Psychiatry, University of Massachusetts Medical School, Worcester, USA; 3 Kyushu University, Faculty of Agriculture, Fukuoka-city, Japan; 4 NINDS, National Institutes of Health, Bethesda, USA

## Abstract

**Background:**

Members of the NET subfamily of zinc-finger proteins are related to the Sp-family of transcription factors and are required during embryogenesis. In particular, Nlz1/Znf703 and Nlz2/Znf503 are required for formation of rhombomere 4 of the vertebrate hindbrain. While NET family proteins have been hypothesized to regulate transcription, it remains unclear if they function as activators or repressors of transcription.

**Results:**

Here we demonstrate that Nlz proteins repress transcription both in cell lines and in developing zebrafish embryos. We first use standard cell culture-based reporter assays to demonstrate that Nlz1/Znf703 represses transcription of a luciferase reporter in four different cell lines. Structure-function analyses and pharmacological inhibition further reveal that Nlz1-mediated repression requires histone deacetylase activity. We next generate a stable transgenic zebrafish reporter line to demonstrate that Nlz1 promotes histone deacetylation at the transgenic promoter and repression of transgene expression during embryogenesis. Lastly, taking a genetic approach we find that endogenous Nlz proteins are required for formation of hindbrain rhombomere 4 during zebrafish embryogenesis by repressing expression of non-rhombomere 4 genes.

**Conclusion:**

We conclude that Nlz1/Znf703 acts as a repressor of transcription and hypothesize that other NET family members function in a similar manner.

## Background

The zebrafish *nlz1/znf703 *and *nlz2/znf503 *genes are closely related to the *Drosophila noc *and *elbow *genes, the *C. elegans tlp-1 *gene and several mammalian genes [1-10]. Together these genes make up a subclass (the NET family) that is related to the Sp family of zinc finger transcription factors (reviewed in [11]). Specifically, NET family proteins share three sequence motifs (a 'buttonhead box', an 'Sp motif' and a C_2_H_2 _zinc finger) with Sp family proteins. While Sp family transcription factors (Sp1–Sp8) are sequence-specific transcription factors acting as activators or repressors depending on the cellular context [12-22], it is unclear if NET proteins play a similar role. For instance, Sp proteins contain three C_2_H_2 _zinc fingers that bind DNA, but NET proteins contain only one zinc finger and this may not be sufficient to bind DNA ([23,24]; reviewed in [25]). Further, the single C_2_H_2 _finger in NET proteins is atypical and is unlikely to bind DNA directly (reviewed in [11]). Accordingly, direct sequence-specific DNA-binding has not been reported for any NET family proteins. Thus, while Sp proteins function as transcription factors, the biochemical activity of NET family proteins is unclear.

Though NET proteins may not bind DNA directly, the available evidence points to their having a role in transcriptional regulation. For instance, Elbow, TLP-1, Nlz1 and Nlz2 are located in the nucleus [2,3,6,7] and Nlz1 must be nuclearly localized to be fully active [3]. Furthermore, gain- and loss-of-function experiments suggest that NET family proteins modulate gene expression during embryogenesis. In particular, expression of *spalt*, a marker of the dorsal tracheal trunk in *Drosophila*, is abolished in response to ectopic expression of *elbow *and is expanded in *elbow *mutants [6]. Similarly, ectopic expression of *nlz1 *or *nlz2 *disrupts *krox20 *expression in rhombomere 3 of the zebrafish hindbrain and leads to an expansion of *hoxb1a *expression from rhombomere 4. Disruption of *nlz *function has the opposite effect, leading to loss of *hoxb1a *expression and expansion of *krox20 *expression [2-4].

While NET proteins appear likely to regulate transcription, it is unclear if they function as activators or repressors or both, as is the case for SP-family proteins. *Drosophila *Elbow is reported to interact with the transcriptional repressor Groucho [6] and both Nlz1 and Nlz2 interact with Groucho as well as with several histone deacetylase (HDAC) co-repressors [2,3]. Against this background, we have hypothesized that NET family proteins act as repressors of transcription during embryogenesis [11], but there is no direct evidence for NET family proteins repressing transcription and it remains plausible that they can function as both activators and repressors.

Here we demonstrate that Nlz proteins repress transcription both in cell lines and in developing zebrafish embryos. We first use standard cell culture-based reporter assays to demonstrate that Nlz1 represses transcription of a luciferase reporter in four different cell lines. We find that this repression requires a domain of Nlz1 that includes the HDAC binding site and that it is blocked by the HDAC inhibitor Trichostatin A, indicating that Nlz1-mediated repression requires HDACs. Next, we generate a stable transgenic zebrafish reporter line and use it to demonstrate that Nlz1 represses a luciferase reporter in the developing embryo. By adapting chromatin immunoprecipitation assays to zebrafish embryos, we further demonstrate that this repression is accompanied by histone deacetylation at the transgene promoter, again consistent with Nlz-mediated repression requiring HDACs. Lastly, we take a genetic approach to examine the function of endogenous Nlz proteins. We find that Nlz proteins are required for formation of hindbrain rhombomere 4 because they repress expression of non-rhombomere 4 genes. We do not find any evidence for Nlz proteins being required as activator during hindbrain formation. We conclude that Nlz proteins act as repressors of transcription and hypothesize that other NET family members function in a similar manner.

## Methods

### Plasmids

The UAS-SV40:Luciferase plasmid was generated by inserting oligonucleotides containing three UAS sites upstream of the SV40 promoter in the pGL3 plasmid (Promega). All Nlz1-GAL4DBD fusion constructs were Myc-tagged at the N-teminus and generated by PCR amplifying the appropriate regions of Nlz1 and subcloning them upstream of the GAL4DBD domain in the pCS2MT expression vector.

### Cell culture, transfection and reporter assay

HeLa, NIH3T3 and SN17 cells were maintained in DMEM containing 10% FBS at 37°C and ZF4 cells were maintained in 1:1 DMEM/F10 with 10% FBS at 28°C. For transfections, 2.5 × 10^5 ^cells were plated in a single well of 6-well plates. The FuGENE6 (Roche) system was used for transfection of HeLa, NIH3T3 and SN17 cells and Effectene (Qiagen) for transfection of ZF4 cells. Luciferase reporter plasmid (1.5 μg, except for ZF4 which was 0.25 μg) was co-transfected with β-galactosidase plasmid (0.5 μg, except for ZF4 which was 0.083 μg) and 20 ng, 100 ng or 500 ng of Gal4DBD or Nlz1-Gal4DBD expression vector (except for ZF4, which was 0.0033, 0.017 and 0.083 ng). The corresponding empty vector was used to adjust the total amount of DNA to 2.5 μg per well (except ZF4, which was 0.42 μg). For TSA experiments, transfected cells were treated with DMSO, 0.1 μM or 0.3 μM TSA starting 24 hrs after transfection. Cells were harvested 48 hrs after transfection. Cells were lysed and analyzed for luciferase and β-galactosidase activities followed by normalization of luciferase activity based on β-galactosidase activity.

### Western blotting

Samples were separated on 12.5% SDS PAGE and transferred to a PVDF membrane. The membrane was incubated in TBS-T Blocking buffer (Tris-Tween plus 5% BSA) for one hour, then with 1 μg/ml anti-Myc antibody in TBS-T 1% BSA at 4°C O/N with gentle shaking. The membrane was then washed with TBS-T, incubated with 1:2000 anti-mouse HRT antibody in 5% skim milk for one hour with gentle agitation, washed again with TBS-T and developed with Supersignal Ultra reagent (Pierce).

### Generation of transgenic UAS-SV40:Luciferase reporter line

The UAS-SV40:Luciferase vector was linearized with NotI. DNA (50 pg) was injected into embryos at the 1-cell stage. Injected embryos were raised to adulthood (F0 generation) and out-crossed to wild type fish. Embryos from each cross were pooled and assayed by PCR (Forward oligo:5-TGC ATC TCA ATT AGT CAG CCA CCA TAG-3; reverse oligo:5-ACC GGA ATG CCA AGC TTT TTG-3) for presence of the transgene. F0 carriers were then out-crossed and the offspring raised to adulthood (F1 generation). F1 fish were genotyped by PCR of fin clip biopsies. Confirmed F1 carriers were used to produce embryos for reporter experiments.

### Promoter assay using TG(UAS-SV40:Luciferase) embryos

F1 TG(UAS-SV40:Luciferase) heterozygous carriers were out crossed and the F2 embryos injected with 400 pg mRNA encoding Gal4DBD or Nlz1-Gal4DBD at the 2–4 cell stage. Injected embryos were raised 11–12 hours (to early somitogenesis stage) and homogenized in 100 μl luciferase lysis buffer (Promega). Each sample was centrifuged and 20 μl of supernatant was used for luciferase assays (Promega). Injection of equivalent amounts of mRNA was confirmed by quantitative PCR.

### Chromatin immunoprecipitation

ChIPs were performed as described previously [26,27] with modifications to allow dissociation of zebrafish embryos into single cells. Specifically, 30 zebrafish embryos were collected at 16hpf, dechorionated and incubated with 40 units of collagenase (Invitrogen) in PBS for 30 minutes at 37°C prior to cross-linking. 1 μl anti-acetyl histone H4 antibody (Upstate) was used for the ChIP. The purified DNA was dissolved in 50 μl Tris-EDTA and 2 μl was used for quantitative PCR. 1% of chromatin taken before immunoprecipitation was used as input. Quantitative PCR was performed with 7.5 μl of QuantiTect SYBR Green PCR mix (Qiagen), 5.5 μl of dH_2_O and 2 μl of DNA with 0.67 μM of the primers (Forward: AAGAAAGGCCCGGCGCCATTCTATCCGCTG, Reverse: GAAGTACTCAGCGTAAGTGATGTCCACCTC). PCR amplification was detected and normalized as a ratio to the input for each sample by using a DNA Engine Opticon (MJ Research).

### Zebrafish lines and in situ hybridization

*vhnf1*^*hi*2169 ^mutants were obtained from N. Hopkins [28]. Nlz loss-of-function embryos were prepared by microinjecting anti-sense morpholino oligos targeting *nlz1 *and *nlz2 *as reported previously [4]. The whole-mount in situ hybridization protocol and probes were described previously [2,3].

## Results

### Nlz1 represses transcription in a diverse set of cell lines

**Figure 1:**
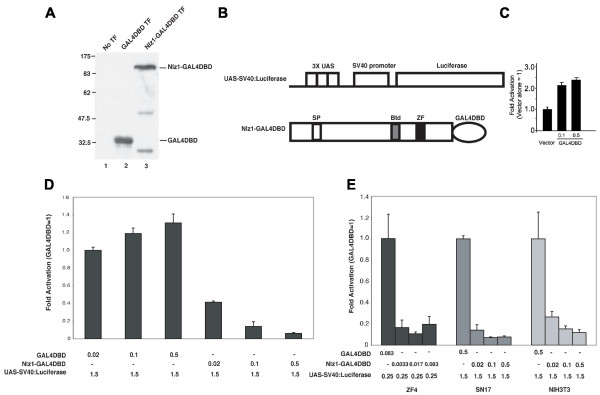
**Nlz1 represses transcription in various cell lines**. **A**. Western blot demonstrating that GAL4DBD (lane 2) and the Nlz1-GAL4DBD fusion protein (lane 3) are well expressed. No TF = untransfected control. **B**. Schematic outline of constructs used. The reporter construct (Top) contains three UAS elements (GAL4 binding sites) upstream of the SV40 promoter and the Luciferase reporter gene. The Nlz1-GAL4DBD construct (bottom) was generated by fusing the GAL4DBD in frame to the C-terminus of full-length zebrafish Nlz1. SP = Sp domain; Btd = buttonhead domain; ZF = C_2_H_2 _zinc finger domain. **C-E**. HeLa cell reporter assays were carried out as described in the Methods section. Luciferase activity was normalized to β-galactosidase activity and the data are presented as fold activation relative to control vector. **C**. The Gal4DBD domain is a weak activator. **D**. Nlz1 represses transcription from the UAS-SV40:Luciferase reporter in HeLa cells. **E**. Nlz1 represses transcription in ZF4, SN17 and NIH3T3 cells.

To determine if Nlz1 acts as a repressor of transcription, we first turned to a cell culture-based reporter assay. Since Nlz1 is unlikely to bind DNA directly, we generated a fusion protein where the DNA-binding domain (DBD) from the yeast GAL4 protein is fused in-frame to the C-terminus of full-length Nlz1. Western blotting experiments demonstrate that the resulting Nlz1-GAL4DBD protein is well expressed in HeLa cells (Fig. 1A). Transcription activity was measured using a reporter plasmid (UAS-SV40:Luciferase) containing 3 GAL4 binding sites (upstream activator sequence; UAS) in front of the SV40 promoter driving luciferase expression (Fig 1B). Transfection of the reporter plasmid alone produces robust luciferase expression in HeLa cells. Co-transfection of the GAL4DBD produces a small, but reproducible increase in luciferase activity (Fig. 1C, D), suggesting that the GAL4DBD has weak activator activity. In contrast, co-transfection of the Nlz1-GAL4DBD fusion protein produces a dose-dependent reduction in luciferase levels (Fig. 1D). While the extent of this repression varies slightly between experiments, we routinely observe up to a 10–40 fold repression (n = 6 experiments). This repression requires recruitment of Nlz1 to DNA, since Nlz1 lacking the GAL4DBD domain does not repress luciferase expression in this assay (not shown). Analyses of Nlz1-GAL4DBD function in ZF4 (zebrafish fibroblast), SN17 (rat neuronal) and NIH3T3 (mouse fibroblast) cell lines also demonstrate robust repression of luciferase activity (Fig. 1E). We conclude that Nlz1 has the ability to repress transcription in cell lines derived from different tissues and various organisms.

### The HDAC binding domain in Nlz1 is required for repression

**Figure 2:**
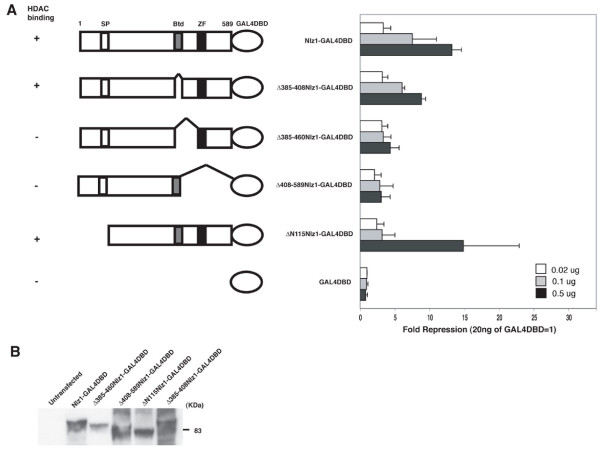
**Nlz1-mediated repression requires an intact HDAC interaction domain**. **A**. Schematic outline of deletion constructs used in reporter assay. SP = Sp domain; Btd = Buttonhead domain; ZF = C_2_H_2 _zinc finger domain. Column at left indicates binding of each construct to HDACs as reported previously [3]. Graph at right indicates the ability of each construct to repress the UAS-SV40:Luciferase reporter in HeLa cells. Transfection was carried out as in Fig. 1 using DNA doses as indicated. Data are presented as fold repression relative to 20 ng of GAL4DBD alone. **B**. Western blot demonstrating that each construct is well expressed in HeLa cells.

We have previously demonstrated that Nlz proteins bind HDAC1 and HDAC2 histone deacetylases [2,3] and HeLa cells are known to express HDACs. Hence, we next used our previous structure-function data to begin testing whether Nlz1 utilizes HDACs to repress transcription in HeLa cells. Specifically, the domain of Nlz1 required for HDAC binding appears to reside between the 'buttonhead box' (Btd) and the C_2_H_2 _zinc finger (ZF), although the buttonhead box may also be required for optimal binding ([2,3]; Summarized in Fig. 2A).

**Figure 3:**
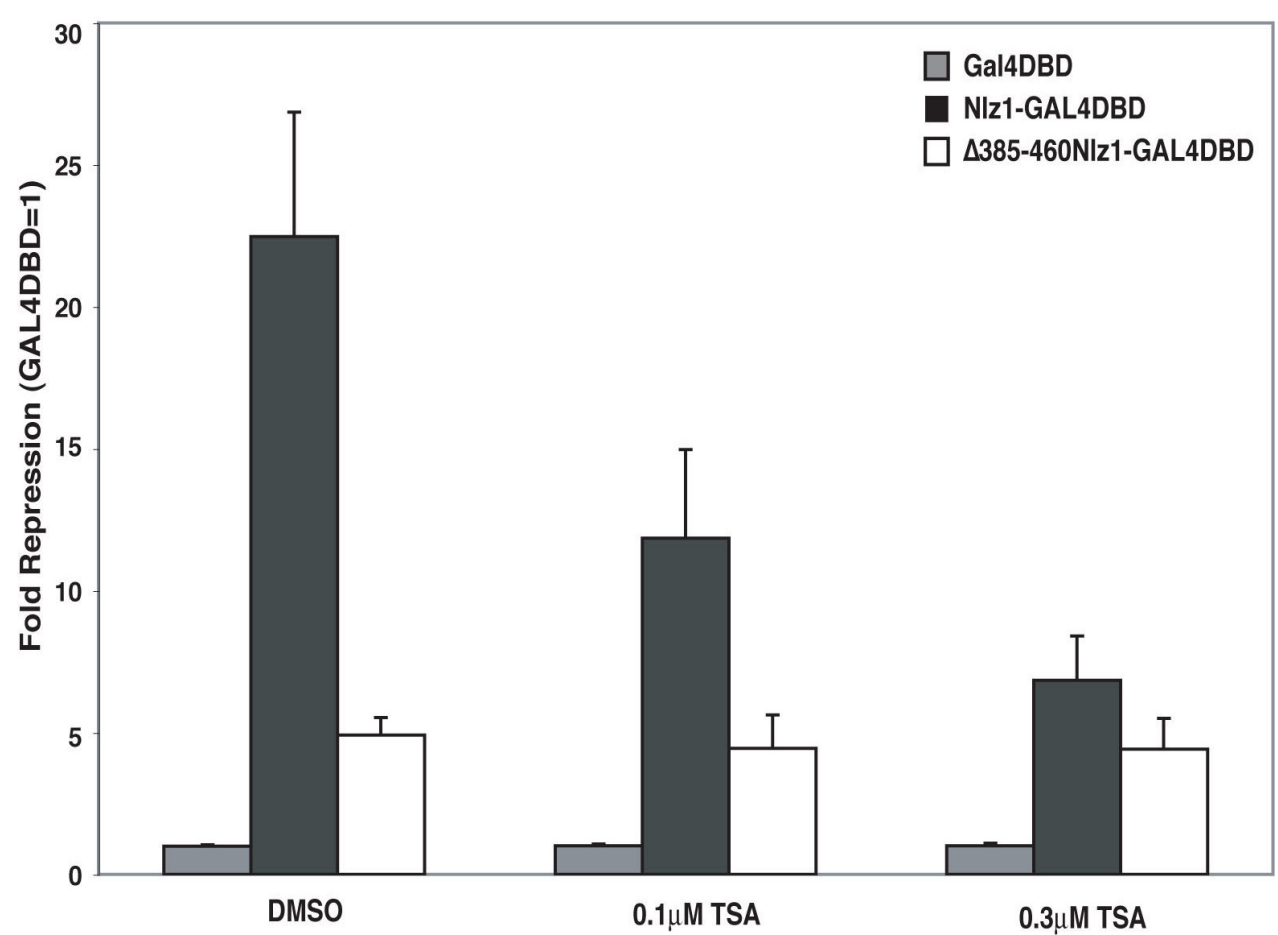
**TSA relieves Nlz1-mediated repression.** HeLa cells were transfected with the UAS-SV40:Luciferase reporter together with GAL4DBD fusion constructs as indicated. Transfected cells were treated with 0.1 μM TSA, 0.3 μM TSA or DMSO as indicated in the graph. Data are expressed as fold repression relative to GAL4DBD alone.

To test which domains of Nlz1 are required for repression in the reporter assay, we fused a series of Nlz1 deletion constructs to the GAL4DBD (Fig. 2A). Western blotting experiments demonstrate that all constructs are well expressed upon transfection into HeLa cells (Fig. 2B). Additionally, the GAL4DBD contains a nuclear localization signal ensuring that all fusion proteins localize to the nucleus (not shown). We next examined each of the deletion constructs for its ability to repress the UAS-SV40:Luciferase reporter in HeLa cells (Fig. 2A). As expected, we find that intact Nlz1-GAL4DBD represses Luciferase expression in a robust, dose-dependent manner. Further, deleting the buttonhead box (Δ385–408) has a minimal effect on HDAC binding (Fig. 2A; [3]) and this construct retains repressor activity (~2 fold less repression than full-length Nlz1; Fig. 2A). In contrast, Nlz1 constructs lacking the HDAC-interaction domain (Δ385–460, Δ408–589) repress the reporter only weakly (~4–5 fold less repression than full-length Nlz1). Together these results demonstrate that the HDAC-interaction domain is required for optimal activity of Nlz1 and are consistent with Nlz1 recruiting HDACs to repress transcription. However, we note some residual repressor activity (~3 fold above GAL4DBD alone) in the two constructs that do not bind HDACs (Δ385–460 and Δ408–589) suggesting that Nlz1 may also repress transcription by a HDAC-independent mechanism.

Nlz family proteins share an N-terminal sequence motif (S-P-L-A-L/M-L-A-A/Q-T-C) with Sp-family proteins (SP; Fig. 2A). While the function of this domain is not fully understood, it has been implicated in regulating the transcription activity of Sp1 [29]. Further, although this motif is not involved in binding to co-repressors, we have shown that it is required for Nlz1 function in zebrafish embryos [2,3]. We therefore tested a construct lacking the N-terminal 115 amino acids, including the Sp motif, in the reporter assay. We find that ΔN115Nlz1-GAL4DBD has robust repressor activity (Fig. 2A), indicating that this domain is not required for Nlz1-mediated repression in HeLa cells (see discussion).

### Nlz1-mediated repression is sensitive to Trichostatin A

To further test whether Nlz1-mediated repression requires HDAC activity, we made use of trichostatin A (TSA), an inhibitor of HDAC activity [30]. For these experiments HeLa cells were transfected with the UAS-SV40:Luciferase reporter together with Nlz1-GAL4DBD, Δ385–460Nlz1-GAL4DBD or GAL4DBD and treated with various concentration of TSA (Fig. 3). In the absence of TSA, the Nlz1-GAL4DBD construct represses luciferase expression ~22 fold, similar to the results in Figs. 1 and 2. However, addition of TSA revealed a dose dependent block of this repression such that at 0.3 μM TSA, we observed only 6–7 fold repression. We were unable to test higher concentrations of TSA since treatment with higher doses was toxic to the cells. However, higher TSA doses may not completely block Nlz1-mediated repression. In particular, the Δ385–460Nlz1-GAL4DBD construct that cannot bind HDACs repressed ~5-fold in both the presence and absence of TSA. Hence, we conclude that HDAC activity is required for optimal Nlz-mediated repression, but our results again suggest an HDAC-independent component of Nlz1-mediated repression.

### Nlz1 represses a UAS-SV40:Luciferase transgene in zebrafish embryos

**Figure 4:**
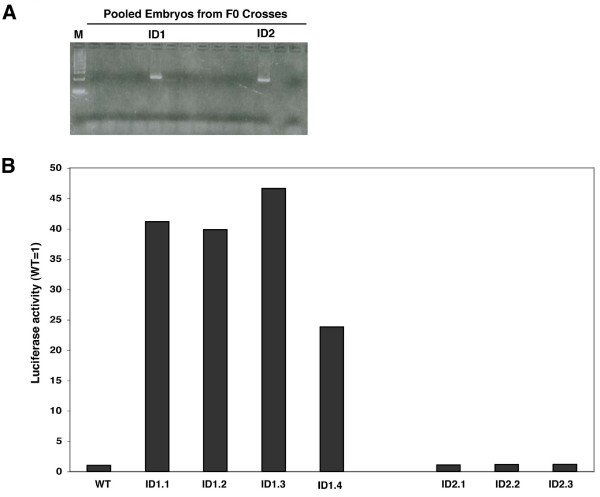
**Derivation of the stable transgenic *TG*(*UAS-SV40:Luciferase) *zebrafish reporter line.A**. Potential F0 founder fish were identified by PCR as outlined in the Methods section. Two founders (ID1 and ID2) were identified. **B**. Expression of the Luciferase reporter in stable transgenic embryos. *TG*(*UAS-SV40:Luciferase) *F1 carriers were out-crossed and their offspring assayed for luciferase activity. Four F1 carriers derived from the ID1 founder (ID1.1, ID1.2, ID1.3 and ID 1.4) produced embryos with robust luciferase activity, while 3 carriers derived from the ID2 founder (ID2.1, ID2.2 and ID2.3) produced embryos lacking luciferase activity.

We next set out to test whether Nlz1 acts as a repressor also in zebrafish embryos. To this end, we first generated a stable *UAS-SV40:Luciferase *transgenic reporter line. Two transgenic carriers were identified (ID1 and ID2; Fig. 4A) and we find that embryo lysates from all ID1-derived animals display robust luciferase activity (Fig. 4B). The similar luciferase expression levels are consistent with all ID1-derived animals containing the same transgene integration event, although this conclusion awaits final confirmation. We refer to this line as *TG(UAS-SV40:Luciferase)*. In contrast, ID2-derived animals do not express luciferase, suggesting that the transgene is not active in this line, perhaps as a result of silencing at the integration site.

**Figure 5:**
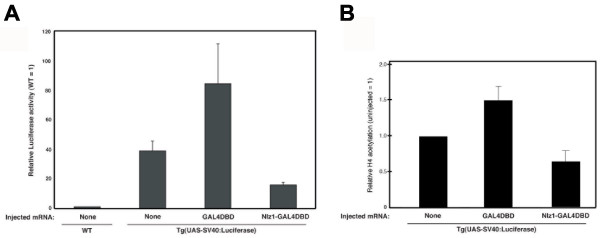
**Nlz1 represses expression of the *luciferase *transgene and promotes histone deacetylation at the transgenic promoter in *TG(UAS-SV40:Luciferase) *embryos.A, B**. *TG(UAS-SV40:Luciferase) *embryos were injected with mRNA encoding GAL4DBD or Nlz1-GAL4DBD as indicated under the graph. Embryos were raised for 12 hours and assayed for luciferase activity (A) or for histone H4 acetylation at the transgenic promoter (B) as described in the Methods section. Data are expressed as fold luciferase activity (A), or fold H4 acetylation (B) relative to un-injected control. A T-test indicates p < 0.0005 for the difference in acetylation level between GAL4DBD- and Nlz1GAL4DBD-injected embryos.

We next used F2 *TG(UAS-SV40:Luciferase) *embryos to test whether Nlz1 acts as a repressor in vivo (Fig. 5A). We find that injection of Nlz1-GAL4DBD mRNA reproducibly represses luciferase activity about 2.5-fold compared to un-injected transgenic embryos. Control injections of GAL4DBD mRNA does not repress expression from the *TG(UAS-SV40:Luciferase) *transgene – instead GAL4DBD leads to ~2-fold activation, similar to the weak activation by GAL4DBD in our cell-based reporter assays (Fig. 1). We also used chromatin immunoprecipitation (ChIP) to determine whether repression of the luciferase transgene is accompanied by reduced histone acetylation at the transgenic promoter (Fig. 5B). We find that histone H4 is deacetylated at the transgenic promoter in embryos expressing Nlz1-GAL4DBD relative to control embryos. In agreement with the apparent ability of the GAL4DBD control construct to activate expression of the luciferase reporter, we observe increased histone H4 acetylation at the transgenic promoter in embryos expressing the GAL4DBD construct. It is possible that higher doses of Nlz1-GAL4DBD mRNA would lead to further histone deacetylation and repression, but we have been unable to test this possibility since we find that higher concentrations of mRNA are toxic to zebrafish embryos. Nevertheless, our results demonstrate that Nlz1 can repress transcription and promote histone deacetylation in zebrafish embryos.

### Nlz proteins are required for rhombomere-restricted expression of gbx1 and vhnf1

**Figure 6:**
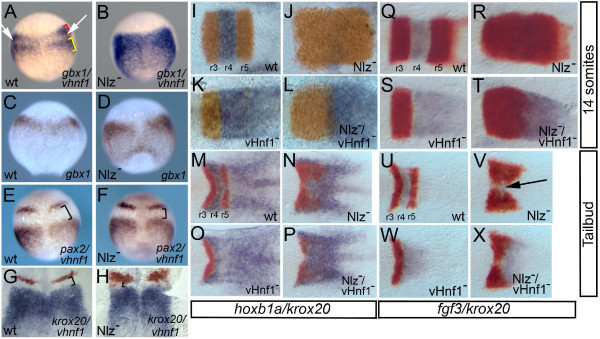
**Nlz proteins act as repressors during zebrafish hindbrain development.A-H**. Nlz proteins repress *vhnf1 *and *gbx1 *expression in rhombomere 4. Wild type embryos (A, C, E, G) or embryos injected with antisense morpholino oligos targeting Nlz1 and Nlz2 (Nlz^-^; B, D, F, H) were assayed by whole mount in situ hybridization for expression of *gbx1*/*vhnf1 *(A, B: *gbx1 *red bracket, *vhnf1 *yellow bracket), *gbx1 *(C, D), *pax2/vhnf1 *(E, F; both detected in purple) and *krox20/vhnf1 *(G, H; *krox20 *detected in red, *vhnf1*detected in blue). White arrows in A and black brackets in E-H indicate gaps in gene expression. **I-X**. Removing *vhnf1 *function restores late gene expression to Nlz^- ^embryos. Wild type (I, M, Q, U), embryos injected with anti-Nlz MO (Nlz^-^; J, N, R, V), *vhnf1 *mutant embryos (K, O, S, W) and *vhnf1 *mutant embryos injected with anti-Nlz MO (Nlz^-^/*vhnf1*^-^; L, P, T, X) were assayed by in situ hybridization for expression of *krox20 */*hoxb1a *(I-P; *krox20 *detected in red, *hoxb1a *detected in blue) or *krox20/fgf3 *(Q-X; *krox20 *detected in red, *fgf3 *detected in blue) at the 14 somite stage (I-L; Q-T) or the tailbud stage (M-P; U-X). Embryos in I-X were dissected and flat-mounted such that only the hindbrain is shown. Anterior is to the top in A-H and to the left in I-X).

To further explore if Nlz proteins act as repressors in vivo, we next turned to Nlz loss-of-function (*nlz*^-^) embryos. During early embryogenesis, the vertebrate hindbrain is transformed from an immature and relatively uniform structure into a structure that is subdivided into a series of rhombomere segments (reviewed in [31]). Nlz1 and Nlz2 proteins are required in this process [2-4], but the effect of disrupting Nlz function (achieved with antisense morpholino oligos [MOs] to Nlz1 and Nlz2, or a Nlz dominant negative construct) has only been assayed for two genes. Specifically, loss of Nlz function leads to expansion of *krox20 *expression from rhombomere 3 (r3) and r5 into r4 and loss of *hoxb1a *expression in r4 [2-4]. Notably, there are genes acting earlier than *krox20 *in r3 and r5. In particular, the *vhnf1 *transcription factor is reported to be the earliest-acting gene in the formation of r5 and more posterior rhombomeres (at least r5 and r6; [28,32-34]), while the *gbx1 *transcription factor acts early in more anterior rhombomeres (r1-r3;[35]). Thus, we examined *vhnf1 *and *gbx1 *expression in *nlz*^-^embryos. As expected, simultaneous detection of *vhnf1 *and *gbx1 *expression by whole mount in situ hybridization reveals a gap corresponding to r4 in wild type embryos (Arrows in Fig. 6A). In contrast, *nlz*^- ^embryos lack such a gap (Fig. 6B). Specifically, the *gbx1 *expression domain expands and low-level *gbx1 *expression appears caudally in *nlz*^- ^embryos (Fig. 6C, D). The *vhnf1 *expression domain also appears to expand as revealed by a reduced gap between *vhnf1 *expression in r5/r6 and *pax2 *expression at the midbrain-hindbrain boundary (Fig. 6E, F), as well as between *vhnf1 *expression in r5/r6 and *krox20 *expression in r3 (Fig. 6G, H). These findings demonstrate that Nlz proteins are required to repress expression of *vhnf1 *and *gbx1 *in r4, but do not reveal whether this effect is direct or indirect.

### Nlz proteins act as repressors in r4

Our results suggest that Nlz proteins are required to repress expression of *vhnf1 *and *gbx1 *in r4. Failure to repress these genes could account for the reported loss of *hoxb1a *expression in r4 of *nlz*^- ^embryos. In particular, *vhnf1 *is known to repress *hoxb1a *expression [28,32-34] and expression of *vhnf1 *in r4 of *nlz*^- ^embryos might therefore suppress *hoxb1a *expression.

We took a genetic approach to determine if Nlz proteins are required in r4 because they repress *vhnf1 *expression. In particular, if *hoxb1a *expression in r4 requires that Nlz proteins repress expression of *vhnf1*, then removing *vhnf1 *function on a *nlz *loss-of-function background might restore *hoxb1a *expression. Put more generally, if expression of *hoxb1a *requires repression of a repressor, then removing both repressors should restore *hoxb1a *expression. As reported, *hoxb1a *expression is lost in *nlz*^- ^embryos (Fig. 6J; [4]) by the 14 somite stage. Strikingly, we find that *hoxb1a *expression is robustly restored when *vhnf1 *function is removed on the *nlz*^- ^background (Fig. 6L). We also note the same behavior for *fgf3 *expression, as *fgf3 *expression is lost in r4 of *nlz*^- ^embryos (Fig. 6R) and restored to *nlz*^-^/*vhnf1*^- ^embryos (Fig. 6T). Thus, when *vhnf1 *is missing, Nlz proteins are not required for *hoxb1a *or *fgf3 *expression – consistent with Nlz proteins acting to repress *vhnf1 *expression. This finding also excludes the possibility that Nlz proteins are required to activate *hoxb1a *or *fgf3 *expression in r4. Notably, since *vhnf1 *function is lacking in r5/r6 of *nlz*^-^/*vhnf1*^- ^embryos, *krox20 *expression is lost from r5 and r4 expression extends into r5/r6 in these embryos, as also observed in *vhnf1*^- ^embryos (Fig. 6K, S; [28,33,34]). Lastly, *nlz*^-^/*vhnf1*^- ^embryos display expanded r3 *krox20 *expression, perhaps as a result of a failure to repress *gbx1 *expression. In summary, our genetic analysis supports a role for Nlz proteins in repressing expression of non-r4 genes.

Rhombomere 4 has been postulated as an important signaling center required for segmentation of the hindbrain [36,37]. Yet, while *nlz*^- ^embryos lack r4, the adjacent r3 and r5 territories appear relatively intact (Fig. 6J, R; [4]). We therefore examined earlier stage embryos and find that *hoxb1a *is robustly expressed in *nlz*^- ^embryos at the tailbud stage (Fig. 6N). Similarly, although *fgf3 *expression is weak in tailbud stage embryos, we routinely observe *fgf3 *expression in the region corresponding to r4 of *nlz*^- ^embryos (arrow in Fig. 6V). Thus, r4 gene expression appears to be initiated in *nlz*^- ^embryos, further supporting our hypothesis that Nlz proteins are not required to induce gene expression in r4, but to repress expression of non-r4 genes.

## Discussion

Based on their sequence relationship to Sp transcription factors and their roles during embryonic development, Nlz proteins and other NET family members have been postulated to regulate transcription. In particular, Nlz proteins share several domains, including a C_2_H_2 _zinc finger, with Sp transcription factors and Nlz proteins localize to the nucleus. Additionally, mis-expression of *nlz1 *or *nlz2 *blocks expression of *krox20 *in rhombomere 3 of the zebrafish hindbrain and permits expansion of r4 gene expression [2]. Conversely, disruption of Nlz function leads to expansion of *krox20 *expression accompanied by loss of *hoxb1a *expression, albeit to various degrees [2-4]. Here we use a combination of biochemical and genetic approaches to demonstrate that Nlz proteins function as repressors, at least in part by recruiting HDACs, and that they act to repress non-r4 genes during zebrafish hindbrain development.

### Biochemical activity of Nlz proteins

Our results demonstrate that Nlz proteins repress transcription of a luciferase reporter gene both in cell lines and in zebrafish embryos. Our structure-function analysis revealed that Nlz proteins require a region containing the HDAC interaction domain for optimal repression activity. In addition, Nlz1-mediated repression in cell lines is blocked by the HDAC antagonist Trichostatin A (TSA) and Nlz1 expression promotes histone deacetylation at a transgenic promoter in zebrafish embryos. These findings suggest that Nlz proteins repress transcription by recruiting HDACs to target promoters. Notably, HDACs are broadly expressed in zebrafish embryos [38], suggesting that they are available for Nlz-mediated repression. Our results also indicate that Nlz proteins may repress transcription partly by an HDAC-independent mechanism. Specifically, we observe residual repressor activity for Nlz constructs that cannot bind HDACs and Nlz1 retains some repressor activity even in the presence of TSA. Although we do not presently know how this residual repressor effect is mediated, our finding that Nlz proteins repress transcription by recruiting HDACs represents an important advance in the understanding of Nlz function.

We have noted that NET family proteins are unlikely to bind DNA directly. These proteins may therefore need to be recruited to DNA by other DNA binding factors, but it is presently unclear how this may be achieved. A potential clue may be provided by our structure-function analysis, which revealed that the N-terminal SP domain (that is conserved between NET and Sp family proteins) is not required for Nlz-mediated repression in cell lines. This is in contrast to our previous analysis in zebrafish embryos, where we found that the Sp domain is required for optimal Nlz1 function [3]. We note that these analyses differ such that our current report utilizes a Nlz1-GAL4DBD fusion protein – where Nlz1 is recruited to DNA via the GAL4 DNA binding domain – whereas our previous work utilized wild type Nlz1. Hence, it is possible that the Sp domain acts as a binding site for a protein that recruits Nlz1 to target promoters in the embryo, although this possibility awaits confirmation by the identification of proteins that bind the Sp motif. The role of this motif in Sp protein function is not clear and it has been ascribed several potential functions (e.g. regulation of protein stability[39]), but there are reports that the Sp motif acts as a protein interaction site [29].

Lastly, while we have not detected any evidence that Nlz proteins can activate transcription, we cannot exclude the possibility that Nlz proteins function as activators in specific cellular contexts. Indeed, some Sp proteins are thought to function as activators or repressors depending on the availability of cofactors (reviewed in [40]). Since such an activator function might be obscured by the high basal activity of the SV40 promoter in our reporter construct, we have tested if Nlz1-GAL4DBD can activate transcription from a reporter construct containing a minimal β-globin promoter, but we have not observed activation of transcription (not shown).

### Biological role of Nlz proteins

Disruption of Nlz function leads to loss of *hoxb1a *expression in r4 and expansion of *krox20 *expression from r3 and r5 of the hindbrain. While *hoxb1a *is one of the earliest-acting genes in r4, there are several genes acting earlier than *krox20 *in r3 and r5. In particular, *vhnf1 *is reported to act very early in the formation of r5 and to be required for *krox20 *expression in r5 [28,33,34], while *gbx1 *acts early in r1-r3 [35]. Hence, we examined expression of *vhnf1 *and *gbx1 *in Nlz loss-of-function embryos and found that expression of these genes expands into r4 – indicating that *gbx1 *and *vhnf1 *expression is normally repressed in r4 by Nlz proteins. Since vHnf1 represses *hoxb1a *expression [28,32-34], we postulated that Nlz proteins might function to repress *vhnf1 *expression in r4 – thereby preventing *vhnf1*-mediated repression of *hoxb1a *expression. In support of this hypothesis we find that removing *vhnf1 *function on a Nlz loss-of-function background restores *hoxb1a *expression, indicating that Nlz proteins are required to repress *vhnf1 *expression in cells that will form r4. At present, we do not know if this repression is direct (i.e. whether Nlz acts at the *vhnf1 *promoter to repress transcription) or if there are additional intervening steps. This issue is complicated by the fact that Nlz proteins may need to be recruited to target promoters by as yet unknown DNA binding proteins (see above), further underscoring that an important next step will be to identify Nlz-binding proteins. We also note that *gbx1 *expression expands throughout the caudal embryo in the absence of Nlz function. This indicates that Nlz may repress gene expression also outside r4, consistent with the broad expression of *nlz *genes at early stages of development.

## Conclusion

We demonstrate that Nlz1 represses transcription of a luciferase reporter in four different cell lines and that this repression requires HDACs. We generate a transgenic zebrafish reporter line and demonstrate that Nlz1 represses transcription and promotes histone deacetylation at the transgene promoter in developing embryos, consistent with Nlz1-mediated repression requiring HDACs also in vivo. Lastly, we take a genetic approach to demonstrate that Nlz proteins are required as repressors, but not activators, of transcription in formation of hindbrain rhombomere 4 during zebrafish embryogenesis. We conclude that Nlz proteins act as repressors of transcription and hypothesize that other NET family members function in a similar manner. Our results also demonstrate the utility of stable transgenic zebrafish-based reporter assays as a complement to cell culture-based reporter assays.

## Authors' contributions

MN carried out the cell-culture reporter assays, generated the TG(UAS-SV40:Luciferase) line, carried out luciferase assays in zebrafish embryos, participated in the ChIP experiments and helped draft the manuscript. S-KC carried out the ChIP experiments and the genetic analysis. APR generated several plasmid constructs and carried out preliminary analyses. PDG helped design the study and interpret the data. CGS conceived of the study and its design, coordinated the study, analyzed the data and wrote the manuscript. All authors read and approved the final manuscript.
